# Adrenal Oncocytic Neoplasm with Paradoxical Loss of Important Mitochondrial Steroidogenic Protein: The 18 kDA Translocator Protein

**DOI:** 10.1155/2017/6734695

**Published:** 2017-11-26

**Authors:** Roberto Ruiz-Cordero, Alia Gupta, Arumugam R. Jayakumar, Gaetano Ciancio, Gunnlaugur Petur Nielsen, Merce Jorda

**Affiliations:** ^1^Department of Hematopathology, University of Texas MD Anderson Cancer Center, Houston, TX 77030, USA; ^2^Department of Pathology, Jackson Memorial Hospital/University of Miami Miller School of Medicine, Miami, FL 33136, USA; ^3^South Florida Foundation for Research and Education Inc., Veterans Affairs Medical Center, Miami, FL 33125, USA; ^4^Department of Surgery, Jackson Memorial Hospital/University of Miami Miller School of Medicine, Miami, FL 33136, USA; ^5^Department of Pathology and Center for Cancer Research, Massachusetts General Hospital, Charlestown, MA 02129, USA; ^6^Department of Urology, Jackson Memorial Hospital/University of Miami Miller School of Medicine, Miami, FL 33136, USA

## Abstract

The adrenal glands produce a variety of hormones that play a key role in the regulation of blood pressure, electrolyte homeostasis, metabolism, immune system suppression, and the body's physiologic response to stress. Adrenal neoplasms can be asymptomatic or can overproduce certain hormones that lead to different clinical manifestations. Oncocytic adrenal neoplasms are infrequent tumors that arise from cells in the adrenal cortex and display a characteristic increase in the number of cytoplasmic mitochondria. Since the rate-limiting step in steroidogenesis includes the transport of cholesterol across the mitochondrial membranes, in part carried out by the 18-kDa translocator protein (TSPO), we assessed the expression of TSPO in a case of adrenal oncocytic neoplasm using residual adrenal gland of the patient as internal control. We observed a significant loss of TSPO immunofluorescence expression in the adrenal oncocytic tumor cells when compared to adjacent normal adrenal tissue. We further confirmed this finding by employing Western blot analysis to semiquantify TSPO expression in tumor and normal adrenal cells. Our findings could suggest a potential role of TSPO in the tumorigenesis of this case of adrenocortical oncocytic neoplasm.

## 1. Introduction

Adrenal oncocytic neoplasms (AON) are infrequent, usually benign, nonfunctional tumors arising in the adrenal cortex that occasionally display borderline or malignant clinical courses. Histologic classification systems (i.e., Weiss system) can usually predict aggressive behavior in regular (nononcocytic) adrenocortical neoplasms; however, histomorphologic features in AON do not always correlate with clinical outcome [1–3].

AON are composed of oncocytes, defined as large eosinophilic cells approximately twice the size of a normal adrenocortical cell with a large central nucleus, a prominent nucleolus, and a characteristic abundant and granular eosinophilic cytoplasm secondary to markedly increased mitochondria [4]. Ultrastructurally, oncocytes are packed with swollen mitochondria. Recent reports strongly support an important role of abnormal steroidogenic events in the pathogenesis of AON [5].

The 18-kDa translocator protein (TSPO) is a ubiquitous mitochondrial nuclear-encoded protein that is upregulated in steroidogenic organs like the adrenal glands and the gonads [6, 7]. Its main function consists in facilitating the migration of cholesterol from the outer to the inner mitochondrial membrane for its conversion into pregnenolone by the cholesterol side-chain cleavage enzyme (CYP11A1) [8, 9]. Thus, transport of cholesterol through the mitochondrial membranes is considered the rate-limiting step in steroidogenesis [8]. Since conspicuous increase in intracytoplasmic mitochondria is sine qua non of AON, we decided to study TSPO expression in one case of AON by means of immunofluorescence. Interestingly, we found a paradoxical loss of TSPO expression in AON cells and confirmed the loss of TPSO expression by Western blot semiquantification.

## 2. Case Presentation

**Figure 1:**
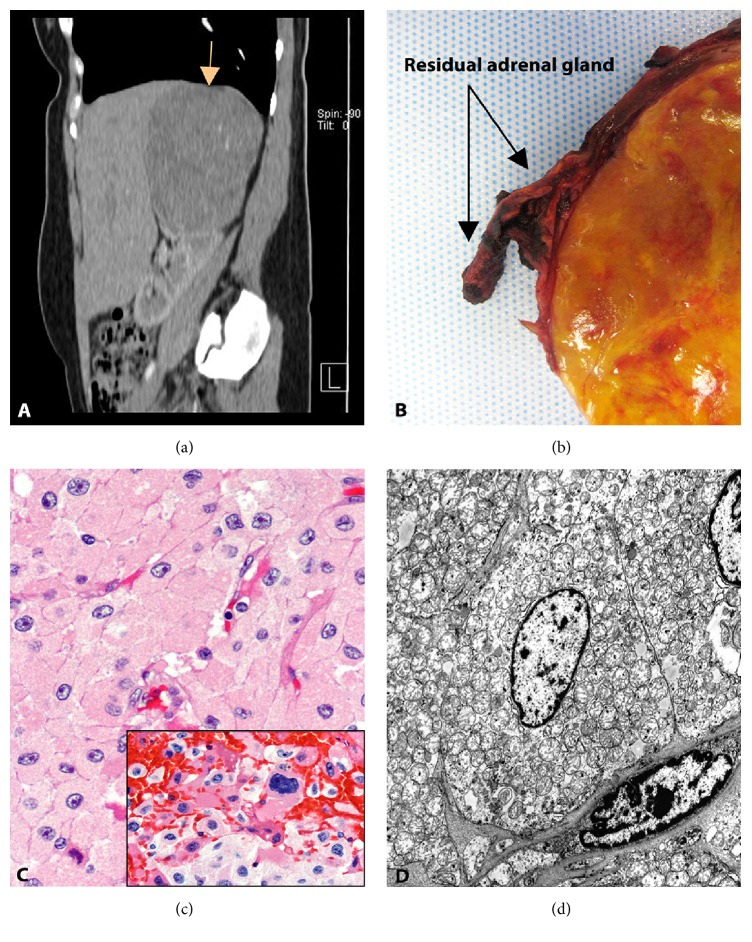
Composite figure illustrating imaging, surgical, histologic, and ultrastructural findings. (a) Sagittal CT scan shows a large ovoid mass (arrow) abutting the liver and the superior pole of the right kidney. (b) Surgical resection specimen highlights the bright yellow tumor parenchyma as well as a portion of the patient's residual adrenal gland (arrows). (c) Microscopic examination of adrenal oncocytic neoplasm composed of large cells with abundant pink granular cytoplasm and irregular nuclei with prominent nucleoli (H&E, 20x). The insert highlights the presence of areas displaying marked nuclear pleomorphism and atypia (H&E, 40x). (d) Transmission electron microscopy illustrating a tumor cell at the center of the image with a large centrally located oval nucleus and abundant mitochondria occupying most of the cytoplasm. H&E: hematoxylin and eosin.

A 49-year-old woman with no significant past medical or surgical history other than sporadic migraines presented to the emergency room at Jackson Memorial Hospital complaining of a 2-week episode of abdominal distention and flank pain. Initial examination revealed an otherwise normal female with vital signs within normal limits and discomfort in the right flank, suspicious for a kidney stone. As part of her initial workup, the patient had an abdominal CT scan that revealed a 15 cm right adrenal mass (Figure 1(a)). No stones or signs of hydronephrosis or pyelonephritis were identified. Laboratory workup, including serum determination of cortisol (5.7 mcg/dL, normal range: 4.3–22.4 mcg/dL at 8 am), aldosterone (<4.0 ng/dL, reference: ≤21 ng/dL), and adrenocorticotropic hormone (12 pg/mL, reference: <47 pg/mL), was unremarkable. The patient underwent surgical excision of the mass. The resected specimen consisted of a well-encapsulated oval mass with a bright golden-yellow parenchyma. The right adrenal gland was found adjacent to the mass (Figure 1(b)). Microscopic examination of the tumor after formalin fixation demonstrated a neoplasm composed of large oncocytic cells (Figure 1(c)) with focal areas of nuclear pleomorphism (Figure 1(c), insert). The presence of increased intracytoplasmic mitochondria was confirmed by electron microscopy (Figure 1(d)). Mitotic figures were not observed. According to the proposed classification by Bisceglia et al. [1], the tumor size and the absence of mitoses, necrosis, capsular, and sinusoidal invasion indicate that this AON could harbor borderline malignant potential. The patient's postsurgical course was unremarkable and no further treatment was required. Currently, four years after surgery, the patient is alive, tumor-free, and in her normal state of health.

### 2.1. TSPO Expression Assessed by Immunofluorescence Is Markedly Decreased in Tumor Cells

**Figure 2:**
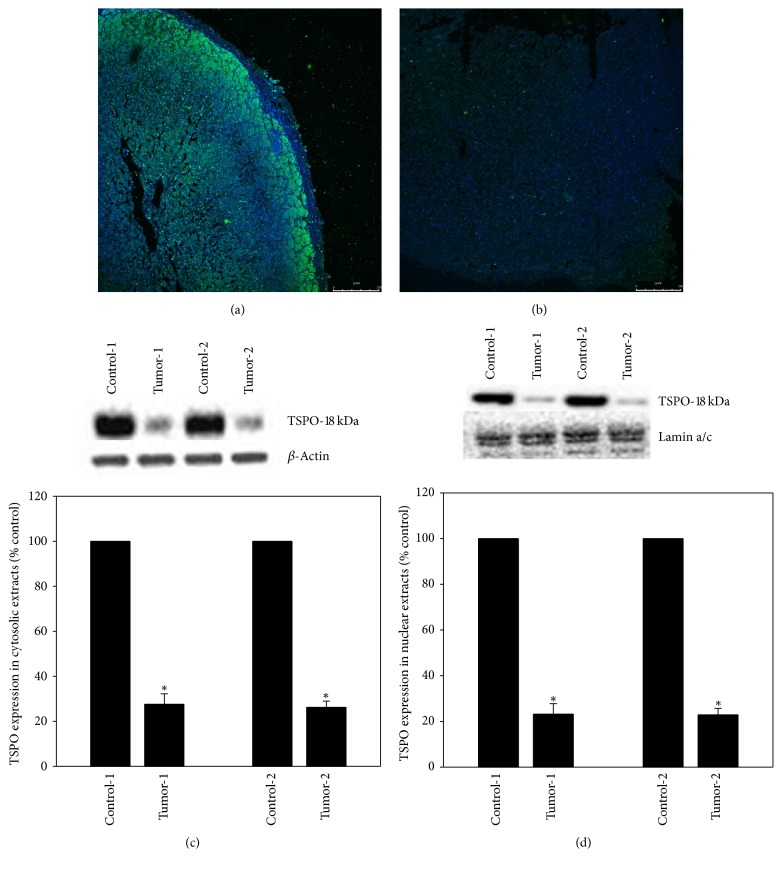
Composite figure illustrating TSPO protein expression findings. (a) Expression of TSPO by immunofluorescence in normal adrenal gland demonstrates diffuse immunofluorescence for TSPO protein, particularly in the zona glomerulosa (IF, 10x). (b) Diffuse loss of TSPO expression assessed by immunofluorescence in adrenal oncocytic tumor cells (IF, 10x). Polyacrylamide gels of the experiments performed in duplicate including normal adrenal cortex from the patient as internal control and tumor, normalized to *β*-actin for cytosolic extracts (c) and lamin a/c for nuclear extracts (d) as housekeeping genes, show a noticeable decrease in the concentration of TSPO in tumor compared to the patient's normal adrenal gland. Western blot semiquantification bar graphs demonstrate 72.4 and 72.8% decrease in cytosolic and 77.1 and 76.8% decrease in nuclear TSPO expression as compared to respective controls. IF: immunofluorescence.

In order to assess the expression of TSPO we obtained additional unstained slides from formalin-fixed paraffin-embedded (FFPE) tissue including a representative section of the tumor with adjacent normal adrenal gland (internal control) of the patient. Two slides were deparaffinized after incubation at room temperature (RT, 24°C) in xylene (twice for 10 minutes). The deparaffinized tissue sections were then rehydrated with a graded series of ethanol (100%, 100%, 70%, 70%, and 50%) and incubated in phosphate buffered saline (PBS) for 15 minutes at RT. After incubation, slides were stained for TSPO by immunofluorescence as previously described [10]. Primary antibody to detect TSPO (FL-169, Santa Cruz Biotechnology, Inc., Dallas, Texas, cat# 20120) was used at 1 : 75 dilution, according to the manufacturer instructions. Fluorescent HRP-conjugated secondary antibody (Alexa Flour-488 goat anti-rabbit IgG (H+L)) was used at 1 : 200 dilutions. The slides were reviewed with a Zeiss LSM510/UV Axiovert 200 M confocal microscope (Carl Zeiss, Peabody, MA, USA). Multiple images captured from tumor cells and normal adrenal gland showed strong immunofluorescence positivity in the normal adjacent adrenal gland (Figure 2(a)) and significant loss of TSPO expression in the tumor cells (Figure 2(b)).

### 2.2. TSPO Semiquantification by Western Blot Confirms Partial Loss of Expression in Tumor Cells

To more precisely evaluate the loss of nuclear and cytoplasmic TSPO expression in tumor cells and confirm the immunofluorescence findings, we dissected mapped tumor and normal adrenal tissue from FFPE unstained slides and performed immunoblots. Briefly, FFPE tissue sections were deparaffinized by incubation at RT in xylene (twice for 10 minutes). The deparaffinized tissue sections were then rehydrated with a graded series of ethanol (100%, 100%, 70%, 70%, and 50%) and incubated at RT in PBS for 15 minutes. After incubation, tumor and normal adrenal tissue were dissected off the slides and placed in two separate plastic tubes. The tubes were pelleted at 16,000 ×g for 5 minutes, and the incubation/centrifugation steps were repeated twice. Tissue samples were briefly air-dried in a fume hood. The cell pellet was resuspended in 200 *μ*l cold buffer A, consisting of 10 mM HEPES (pH 7.9), 10 mM KCl, 0.1 mM EDTA, 0.1 mM EGTA, 1 *μ*M dithiothreitol (DTT), and a complete protease inhibitor cocktail (Roche, Mannheim, Germany). The pellet was then incubated on ice for 15 minutes to allow cells to swell, after which 15 *μ*l of 10% NP-40 was added, and the sample was vortexed thoroughly for 40 seconds and centrifuged at 3,000 rpm for 3 minutes at 4°C. The resulting supernatant was used for cytosolic TSPO measurement (equal amount of protein, 12.4 *μ*g was loaded on an SDS-polyacrylamide gel and Western blot analysis with TSPO antibody was performed as described previously [11]) and the pellet (nuclear fraction) was resuspended in 30 *μ*l cold buffer B consisting of 20 mM HEPES (pH 7.9), 0.4 M NaCl, 1 mM EDTA, 1 mM EGTA, 1 *μ*M DTT, and protease inhibitors. The pellet was then incubated on ice and vortexed for 15 seconds every 2 min for up to 15 min. The nuclear extract was then centrifuged at 13,000 rpm for 5 minutes at 4°C. Equal amounts of protein (21.6 *μ*g) from the supernatant (containing the nuclear extract) were loaded and Western blot analysis with TSPO antibody was performed as described above. The quality of the nuclear extract was analyzed by propidium iodide staining, which indicated a purity of 92–96%. Primary TSPO antibody (Santa Cruz Biotechnology, Inc., Dallas, Texas, cat# 20120) was used at 1 : 1000 dilution. Beta actin (ACTBD11B7, sc-81178, Santa Cruz Biotechnology, Dallas, TX, USA) and lamin a/c (Cell Signaling Technology, Beverly, MA, USA) antibodies were used at 1 : 5000 and 1 : 750, respectively. Anti-rabbit and anti-mouse secondary antibodies (Vector Laboratories, Burlingame, CA, USA) were used at 1 : 3000 dilution. Optical density of the bands was determined with the Chemi-Imager (Alpha Innotech, San Leandro, CA, USA) digital imaging system and the results were quantified with the Sigma Scan Pro (Jandell Scientific, San Jose, CA, USA) program as a proportion of the signal of housekeeping protein bands (lamin a/c and *β*-actin, nuclear and cytosolic markers, resp.). The experiment was performed using 4 different tissue sections from the same sample and the mean intensity of Western blot bands was subjected to Tukey's multiple comparison test. Statistical significance was set at *p* value = 0.05. As illustrated in Figures 2(a) and 2(b) (cytosolic fraction) and Figures 2(c) and 2(d) (nuclear fraction), the representative semiquantitative immunoblots from two different tumor sections show a significant decrease in TSPO expression in the tumor sample of 72.4 and 72.8% decrease in the cytosol and 77.1 and 76.8% decrease in the nuclear fraction as compared to respective controls (*p* = 0.03).

## 3. Discussion

The Weiss classification system for adrenocortical neoplasms has been widely adopted and used to distinguish benign from malignant tumors based on major (high mitotic rate, atypical mitoses, and lymphovascular invasion) and minor criteria (large-size and increased weight, necrosis, capsular invasion, and sinusoidal invasion) [3]. The presence of one major criterion indicates malignancy, 1 to 4 minor criteria indicate uncertain malignant potential (borderline), and the absence of all major and minor criteria suggests a benign clinical behavior [3]. While this classification system has been useful in accurately predicting the biologic behavior of conventional (nononcocytic) adrenocortical tumors, its use in the setting of AON is questionable. In a series of 10 cases of AON, the Weiss system criteria were reviewed and modified to assess its possible application to the oncocytic tumor variant. Using this new grading system, 1 of the 10 cases had to be revised to a final interpretation of malignant after tumor recurrence [1]. In terms of imaging studies, a cutoff below 4-5 cm in tumor size is used to suggest a benign behavior [12]. In cases of oncocytic neoplasms, however, the size of the mass has not demonstrated to reliably predict tumor behavior. Some nonspecific findings such as fat concentration (almost all malignant lesions are lipid poor) and lower attenuation (10 Hounsfield units or less) on CT scan have proven to be helpful in making this differentiation [12, 13].

It has been extensively documented that these oncocytic tumors display characteristic eosinophilic staining secondary to the accumulation of mitochondria, which may occupy up to 60% of the cytoplasm [4]. The increased concentration of mitochondria is accompanied by a gradual compression and sometimes disappearance of other cytoplasmic organelles [14]. Because of the rarity of this type of adrenocortical tumors and because immunohistochemical studies were not consistently performed in most of the reported cases, their immunophenotypic profile has not been completely characterized [4, 15]. Nevertheless, studied cases demonstrate diffuse positivity for vimentin, melan-A, synaptophysin, and inhibin, while S-100 and chromogranin have been consistently negative [15]. In some cases, immunopositivity with an anti-mitochondrial antibody has been used to corroborate that the tumors are truly oncocytic [16].

TSPO is found in the outer mitochondrial membrane of almost every tissue in the body [17]. It is part of a complex of proteins (i.e., StAR, PKA, ACBD3, and VDAC1) that function together by forming a tansduceosome that facilitates cholesterol transport from the outer to the inner mitochondrial membrane for its conversion into pregnenolone by cholesterol side-chain cleavage cytochrome P450 enzyme CYP11A1 [8, 9]. Recently, there has been controversy regarding the critical role that TSPO plays in cellular homeostasis and steroidogenesis. While some authors initially suggested a crucial role based on experiments with TSPO knockout mice whose embryos did not survive, others have replicated similar experiments with different results [18–23]. Nevertheless, in the realm of cancer, several studies have found an increased TSPO protein expression in cancer cell lines and in tumor biopsies of colon, breast, and prostate [19–21]. Moreover, in the particular case of prostatic adenocarcinoma, TSPO protein expression was the highest in metastatic prostate cancer samples where increased expression also correlated with disease progression [19]. More recently, TSPO has been shown to be part of the mitochondria-to-nucleus signaling pathway that modulates nuclear gene expression and TSPO levels have been directly correlated with increased tumorigenicity and/or malignancy, probably as a mechanism to promote apoptosis and reduce tumorigenicity, thereby suggesting that benign tumors do not have increased TSPO levels [6, 24–26]. However, we have observed conventional adrenocortical carcinomas with a wide range of TSPO expression, ranging from no expression to markedly increased TSPO expression [27]. Batarseh and Papadopoulos [17] consider* TSPO* as a highly conserved, housekeeping gene that is expected to remain permanently activated. Recently,* TSPO* gene has been suggested as a novel target for cancer chemotherapy [22]. It is possible that an altered regulation of the numerous cellular processes associated with mitochondria and cholesterol transport could partially be responsible for the unrestrained growth of tumors by mechanisms that remain unknown [17, 19, 23, 28–33].

While previous studies [32] have identified variable TSPO expression in adrenocortical and other tumors, our study represents the first description of TSPO expression in one AON. The present case is unusual and interesting because TSPO expression was paradoxically lost in the mitochondria of the tumor cells when assessed by immunofluorescence in comparison to the patient's normal adrenal gland. When these findings were further explored by direct semiquantification of the presence of protein by Western blot, up to a 77% decrease in expression was confirmed between the tumor and the patient's normal adrenal cells in both cytoplasm and nucleus. While our findings in this one particular case could represent a coincidence, we believe that the fact that a tissue section including the patient's normal adrenal gland adjacent to the tumor used to perform all tests argues otherwise, particularly, because deparaffinization, immunofluorescence, and Western blotting were performed on tumor and normal cells at the same time on the same slide in several occasions. Furthermore, preliminary results on the expression of TSPO in adrenocortical neoplasms in a study performed by our group employing immunohistochemistry demonstrated variable TSPO expression in tumors arising in the adrenal cortex, particularly adrenocortical carcinomas [27].

It is also possible that the increase in mitochondria could be a compensatory phenomenon that could be in part associated with tumor growth. While our findings could indicate that loss of TSPO expression could play a role in the tumorigenesis of this case of adrenocortical oncocytic neoplasm potentially related to defective steroid biosynthesis, additional studies including a larger number of cases of AON are necessary to validate these findings and to determine the role of TSPO in the pathogenesis of adrenocortical neoplasms including AON.
